# Supporting optimization of thick seam roadway with top coal based on orthogonal matrix analysis

**DOI:** 10.1038/s41598-023-27817-8

**Published:** 2023-01-17

**Authors:** Ce Jia, Sheng Li, Chaojun Fan, Mingkun Luo, Lijun Zhou, Ziang Pu, Lei Yang

**Affiliations:** 1 College of Mining, Liaoning Technical University, Fuxin, 123000 China; 2 Zhangcun Coal Mine, Shanxi Lu’an Chemical (Group) Co., Ltd., Changzhi, 046299 China; 3 College of Safety Science and Engineering, Liaoning Technical University, Huludao, 125105 China

**Keywords:** Civil engineering, Coal

## Abstract

Aiming at the problem of large deformation and difficulty in surrounding rock control of the top coal roadway in thick seam, theoretical analysis, theoretical analysis, numerical simulation, orthogonal matrix analysis and other methods were used to study the roof deformation and support parameter optimization of the top coal roadway in thick seam. Firstly, the structural model and roof mechanical model of the top coal roadway in thick seam were established, and the deformation coefficient *T*_*K*_ was defined based on the relationship between curvature radius and bending moment, maximum bending moment and ultimate tensile strength of beam. According to the ratio of deformation rate between *T*_*K*_ and beam to determine the roof deformation mode of top coal roadway, the discriminant conditions of roadway roof stability under two deformation conditions are obtained. Due to the characteristics of serious coal-rock fragmentation, large roof deformation, and integration of top coal and side coal. Therefore, the combined support method of “high prestressed long and short anchor cables” is proposed by double arch bearing structure control technology. Finally, based on the orthogonal matrix analysis method of supporting parameters optimization of the top coal roadway in thick seam, the analysis amount of supporting scheme is significantly reduced, the comprehensive evaluation of multi-factor and multi-supporting effect of roadway support is realized, and the optimal supporting scheme is obtained. Compared with the surrounding rock of the roadway without support, the deformation of the roof is reduced by 27.27%, the deformation of the two sides is reduced by 45.24%, and the tensile failure volume is reduced by 54.66%. The top coal roadway in thick seam has been effectively controlled, which provides guarantee for high yield and high efficiency of the mine.

## Introduction

The thickness of the coal seam is more than 3.5 m, which is called the thick coal seam^1^, and it is the main mineable coal seam to achieve high production and high efficiency in modern Chinese mines^2^. According to incomplete statistics, about 46% of China's coal resource reserves and production come from the mining of thick coal seams^3^. Due to the large thickness of the coal seam, most roadways are excavated along the coal seam floor, leaving top coal. Compared with the rock stratum roof, the reserved top coal has serious fragmentation and poor mechanical properties^4^. As a result, the surrounding rock of the top coal roadway in thick seam is more difficult to control than the general roadway surrounding rock, and the supporting system is complicated, which hinders the efficient and rapid excavation of the mine^5,6^.

Presently, domestic and foreign scholars have conducted relevant research and exploration on problems such as large deformation of the top coal roadway in the thick seam and control of the surrounding rock^7–9^. Kang^10^ and other scholars believe that the roof of the top coal roadway belongs to the composite roof. Dong^11^ obtained the support form and support parameters of the composite roof by using RFPA^2D^ software simulation. Hong^12^ used FLAC^3D^ numerical simulation software to analyze the influence of bolt preload and spacing on the anchoring strength of surrounding rock. Wang^13^ used FLAC^3D^ software and orthogonal experiments to study the influencing factors of the instability and failure of the composite roof. Yu^14^ studied the field structure of weak surrounding rock through numerical simulation, orthogonal tests, field observation, and optimization of combined support parameters. Wang^15^ used a combination of similar materials and numerical simulation to predict the subsidence of the composite roof. Li^16^ studied the instability mechanism of soft rock composite layered roof through theoretical analysis. Yu^17^ derived the deflection of each layer of the composite roof based on the infinite-length plate theory and determined the critical load discriminant of each layer. Xie^18^ studied the support principle of compound roof roadway based on the energy balance theory. Li^19^ proposed a multi-stage prestressed load-bearing arch composed of high prestressed anchor cables, which is a superposition and coupled support technology of large and small structures according to the failure characteristics of the soft rock composite roof. M. M. Murphy^20^ studied the failure characteristics of different weak shale roofs and proposed corresponding control methods. Fu^21^ proposed long and short anchor cable layer control technology for composite soft rock roadway. Wang^22^ established a layered simplified mechanical model of coal roadway based on elastic foundation beam and essential layer theory. However, the above research did not qualitatively analyze the discriminant conditions of roof deformation and failure of supporting roof coal roadway and did not consider multi-factors and comprehensive evaluation of multi-support effects. Therefore, the deformation of the roadway and the optimization of supporting parameters for the top coal roadway in the thick seam have not been studied in depth. The above scholars have mainly studied the control of composite roof from two aspects of mining influence and mechanical properties of surrounding rock. However, the impact of mining is short-lived. This paper focuses on the optimization of rock support parameters for top coal roadways under specific surrounding rock geological conditions.

In this paper, the structural model and the roof mechanics model of the top coal roadway in a thick seam are established. Based on the relationship between the radius of curvature and the bending moment of the beam, the maximum bending moment and the ultimate tensile strength of the beam, the determination conditions for the roof deformation mode of the roof-supporting coal roadway and the stability of the corresponding roadway roof are obtained. Based on the deformation and failure characteristics of the top coal roadway, the control technology of the double arch bearing structure with high prestressed long and short anchor cables is obtained. An orthogonal matrix analysis method for optimization of roadway support parameters in thick coal seam supporting top coal roadway is proposed, which significantly reduces the amount of analysis of support schemes and realizes comprehensive evaluation of multi-factor and multi-support effects of roadway support.

## Surrounding rock deformation and control principle of the top coal roadway in thick seam

### Mechanical model of roadway roof of the top coal roadway in thick seam

**Figure 1:**
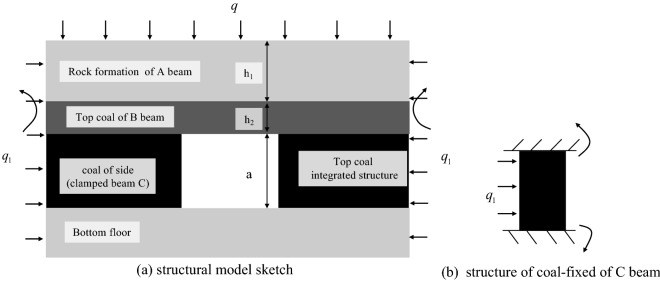
Structural model of the top coal roadway in thick seam.

**Figure 2:**
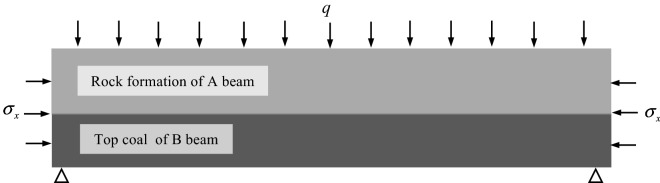
Mechanical model of the roof of the top coal roadway in thick seam.

The average thickness of the mineable coal seam in Zhangcun Coal Mine is 5.69 m. Due to the large thickness of the coal seam, the great majority of the coal mine roadways are mainly supported roof coal roadways with top coal reserved along the bottom. When the thick coal seam is mined, the degree of coal fragmentation is severe, and the roof of the top-supporting coal roadway is challenging to control, which is prone to safety accidents. Based on the engineering geological background of Zhangcun Coal mine, the mechanical model of a thick coal seam supporting roadway roof is established. According to the field engineering problems, the following assumptions were made on the model to analyze its stability: (1) The coal and rock bodies at the roof of the roadway are considered beam A and beam B, which are homogeneous elastic structures. (2) An integrated structure is formed by beam A. The clamped beam C generates a bending moment under the horizontal force *q*_*1*_, as shown in Fig. 1a. (3) Under the action of the horizontal force *q*_*1*_, the clamped beam C will generate a bending moment between the beam B and the bottom floor, as shown in Fig. 1b. (4) The stiffness of beam A is greater than that of beam B, and beam A is the main load-bearing body. (5) There is a cohesive force between beam A and beam B, which both deform synergistically. In the actual strike length of transport roadway 2606, unit length was selected for study, and the beam structure of beam A and beam B with the fixed beam C as the fulcrum is shown in Fig. 2.

According to the previous study^23^, the relationship between the radius of curvature and the bending moment of the beam and the moment of inertia of the beam are expressed as follows:

1 $$\left\{ \begin{gathered} \frac{1}{{\rho_{1} }} = \frac{{M_{1} }}{{E_{1} I_{1} }} \hfill \\ \frac{1}{{\rho_{2} }} = \frac{{M_{2} }}{{E_{2} I_{2} }} \hfill \\ I = \frac{{bh^{3} }}{12} \hfill \\ \end{gathered} \right.$$

where *E*_*1*_ is the elastic modulus of beam A, MPa; *E*_*2*_ is the elastic modulus of beam B, MPa; *M*_*1*_ is the maximum bending moment of beam A, kN·m; *M*_*2*_ is the maximum bending moment of beam B, kN m; *ρ*_*1*_ is the radius of curvature of beam A, m; *ρ*_*2*_ is the radius of curvature of beam B, m; *I* is the moment of inertia of the beam, m^4^; *h* is the height of beam A, m; *b* is the cross-sectional width of the beam, taking 1 m.

According to the mechanics of materials, the maximum bending moment of the two can be expressed as^24^:

2 $$\left\{ \begin{gathered} M_{1} = \frac{{q_{m} l^{2} }}{4} - \frac{{q_{1} a^{2} }}{12} \hfill \\ M_{2} = \frac{{\left( {q + q_{y} } \right)l^{2} }}{4} \hfill \\ \end{gathered} \right.$$

where *q*_*y*_ is the load caused by the self-weight of beam A, kN/m; *q*_*m*_ is the load caused by the self-weight of beam B, kN/m; *q*_*l*_ is the uniform load generated in the horizontal direction, kN/m; *q* is the overlying load of beam A, kN/m.

According to the above analysis, the deformation coefficient *Tk* between beams A and B is defined as follows^25^:

3 $$T_{k} = \frac{{E_{1} }}{{E_{2} }}$$

When the beams A and B are under the combined effect of stress, the maximum tensile stress of the beam structure is^26^:

4 $$\sigma_{\max } = \frac{M}{Wi} - \sigma_{x}$$

where *σ*_*max*_ is the ultimate tensile strength of the beam, MPa; *σx* is the horizontal stress of surrounding rock, MPa; *W*_*i*_ is the bending section coefficient of beam, and its value is bh^2^/6 and M^3^.

When the sinking speed of beam A is lower than the sinking speed of beam B, the curvature radius *ρ*_*1*_ of beam A is less than the radius of curvature *ρ*_*2*_ of beam B, and the roof of the top coal roadway is deformed by layer separation. Simultaneous formulas (1), (2), (3) get the following relational formula:

5 $$T_{k} < \frac{{h_{2}^{3} }}{{h_{1}^{3} }} \cdot \frac{{3q_{{m{ }}} l^{2} - q_{1} a^{2} }}{{3\left( {q + q_{y} } \right)l^{2} }}$$

The compressive strength that the coal rock mass itself can bear is greater than the tensile strength. Therefore, the ultimate tensile strength of beams A and B is the condition for judging the stability of the roof. When the roof of the top coal roadway is deformed by delamination, the conditions for determining the stability of the roadway roof by combining formulas (2) and (4) are:

6 $$\left\{ {\begin{array}{*{20}l} {\sigma_{{A{ }\max }} = \frac{{3\left( {q + q_{{y{ }}} } \right)l^{2} }}{{2h_{2}^{2} }} - \sigma_{x} < \left[ {\sigma_{A} } \right]} \hfill \\ {\sigma_{{B{ }\max }} = \frac{{3q_{m} l^{2} - q_{1} a^{2} }}{{2h_{1}^{2} }} - \sigma_{x} < \left[ {\sigma_{B} } \right]} \hfill \\ \end{array} } \right.$$

When the subsidence rate ratio of beam B and beam A is less than the deformation coefficient, the top coal roadway will undergo large synergistic deformation. Simultaneous formulas (1), (2), (3) get the following relational formula:

7 $$T_{k} > \frac{{h_{2}^{3} }}{{h_{1}^{3} }} \cdot \frac{{3q_{{m{ }}} l^{2} - q_{1} a^{2} }}{{3\left( {q + q_{y} } \right)l^{2} }} \,$$

When the roof of the top coal roadway has a large collaborative deformation, the A beam and the B beam form a superimposed beam. At this time, the stability of the roof depends on the ultimate tensile strength of the B beam, and the judgment conditions for the stability of the roadway roof are:

8 $$\sigma_{{B{ }\max }} = \frac{{E_{2} h_{2} \left( {ql^{2} - 2q_{1} a^{2} } \right)}}{{4k\left( {E_{1} h_{1}^{3} + E_{2} h_{2}^{3} } \right)}} - \sigma_{x} < \left[ {\sigma_{B} } \right]$$

**Figure 3:**
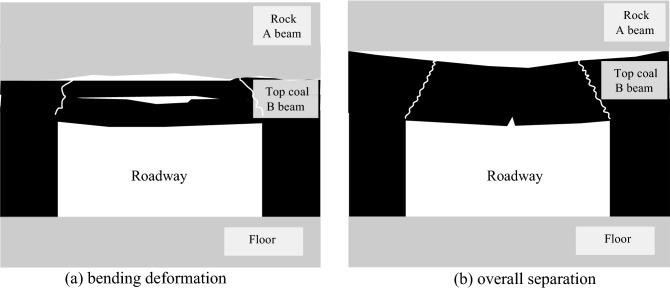
Deformation mode of the roof by top coal roadway in thick seam.

It can be seen from the above analysis that there are two main deformation and failure modes for the roof of the top coal roadway in thick seam. When the deformation between beam A and beam B satisfies Eq. (5), a large synergistic deformation occurs between them, as shown in Fig. 3a. Tensile failure occurs on the roof of the roadway. When the criterion (6) is exceeded, the roof deforms greatly, and the influence range is wide. Due to the large deformation of the roof, the anchor rod and the anchor cable were broken and failed. When the deformation of beam A and beam B satisfies Eq. (7), the delamination occurs between them, as shown in Fig. 3b. When the roadway roof exceeds the judgment condition (8), the separation and sliding between the rock layer and the coal seam occur, and the separation distance increases, resulting in shear deformation of the anchor rod and the anchor cable.

### Combined control technology of long and short anchor cables on roof of top coal roadway in thick seam

**Figure 4:**
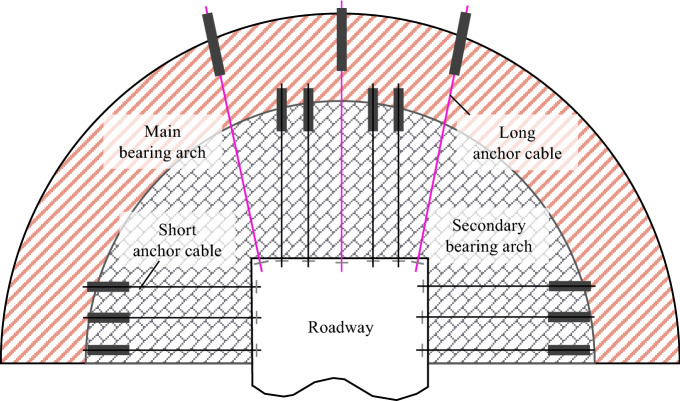
High prestressed long and short anchor cable double arch bearing structure model.

For the top coal roadway in the thick seam, the roof support is significant for the stability of the entire roadway due to the integration of the top coal and the side coal. In summary, the support of the top coal roadway in thick seam should be considered: the high-strength support structure is adopted, and the high-strength support can carry the large coordinative deformation and delamination deformation of the roof. The high prestressed support structure is adopted. Due to the thick coal seam mining, the coal and rock mass is seriously broken, and the high prestressed support structure can carry out effective prestress diffusion and improve the stress state of the surrounding rock. Increase the scope of the anchorage area and improve the bearing capacity of the surrounding rock in the anchorage area. The top coal and the side coal are integrated, the principle of the same treatment of the top and the gang is adopted, and the roof and the gang part reinforcement is also considered. Therefore, the high prestressed long and short anchor cable double arch bearing structure is proposed to control the stability of the top coal roadway in the thick seam, as shown in Fig. 4.

According to Reference^27^, the bearing capacity of anchorage balanced arch is:

9 $$q = \frac{Q(1 + \sin \theta )(l - D)}{{D^{2} (1 - \sin \theta )(R + l - D)}}$$

where *q* is the bearing capacity of the arch, kN; *D* is the bolt or anchor cable spacing, m; *Q* is the bolt or anchor cable preload, kN; *θ* is internal friction angle of coal and rock mass, (°); *l* is the length of the bolt or anchor cable, m; *R* is the half the roadway span, m.

According to Eq. (9), when the mechanical conditions of the roadway surrounding rock and the size of the roadway cross-section are specific, the anchor cable preload, length, and spacing are the main factors affecting the support effect. Consequently, the stability of the surrounding rock can be controlled effectively with the accurate and reasonable design of the three support parameters.

## Numerical simulation analysis of the effect of high prestressed long and short anchor cable support of the top coal roadway in thick seam

**Figure 5:**
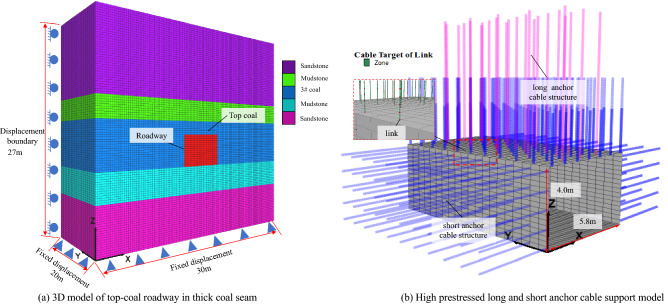
3D model and supporting structure of the top coal roadway in thick seam.

Relying on the geological engineering background of the Zhangcun coal mine, a three-dimensional model with a size of 30 m × 15 m × 27 m (length × width × height) was constructed. The roadway is excavated along the 3# coal seam floor, and the cross-section of the roadway is rectangular, and the cross-sectional size is 5.8 m × 4 m (width × height). The Moore-Coulomb constitutive model was used for this simulation, and the model failure was consistent with the Moore-Coulomb failure criterion^28^. In order to simulate the actual geological environment, the surrounding and bottom surfaces of the model adopt normal displacement constraints, and the upper boundary is free and bears the uniform load acting on the boundary by the overlying rock. The upper boundary load of the model is 10 MPa, as shown in Fig. 5a. The lithological parameters used in the 3D model cover the range of literature^29,30^, as shown in Table 1. Long and short anchor cable supports are simulated by the built-in cable structural elements of FLAC^3D^ software^31^. The anchor cable structural element and the solid element are connected by link, rigid constraints are applied to the nodes, and pallets are simulated to realize the combined support of high prestressed long and short anchor cables in the roadway of the thick coal seam supporting the roof, as shown in Fig. 5b. The parameters used for the anchor cable are shown in Table 2.

**Table 1:** Rock mechanics parameter.

Name	Bulk density (g·cm^−3^)	Bulk modulus (GPa)	Shear modulus (GPa)	Cohesion (MPa)	Internal friction angle (°)	Tensile strength (MPa)
Basic roof	2873	1.84	0.80	2.3	28	2.0
Immediate roof	2487	1.5	1.4	1.02	33	1.8
3#coal	1380	4.91	2.01	2.7	32	0.15
Immediate floor	2483	1.84	0.80	2.3	28	2.0
Basic floor	2460	1.79	0.97	1.9	30	1.6

**Table 2:** Parameters of bolt element.

Name	Young's modulus (Pa)	Elastic modulus (N·m^−2^)	Compressive strength (MPa)	Tensile strength (MPa)	Cross sectional area (m^2^)
Anchor cable	4e^8^	2e^11^	3.9e^2^	1.32e^3^	3.14e^−4^

### Support optimization orthogonal combination design

The stability of the roadway is controlled by the double arch bearing structure with high prestressed long and short anchor cables, but many parameters affect the support effect. Considering the support effect and economic cost, determining which parameters are the main factors affecting the stability of the surrounding rock is an essential link in the design of the support scheme. Therefore, the orthogonal experiment method can reduce the large number of experiments. According to the above formula (9), it can be seen that the three parameters of anchor cable prestress, anchor cable length, and anchor cable spacing are the main factors affecting the support effect. Under the premise of satisfying the factors and levels, a table of factors and levels is made, as shown in Table 3. In order to ensure the error analysis of the experiment, an empty column is set up, which is not listed in the table, and the L9(3^4^) table is made according to the principle of orthogonal experimental design, as shown in Table 4. According to the orthogonal experimental design scheme, FLAC^3D^ software was used to obtain the orthogonal experimental range analysis, as shown in Table 5.

**Table 3:** Factors and levels table.

Level	Factor		
	Anchor cable preload (A)/kN	Anchor cable length (B)/m	Anchor cable spacing (C)/m
1	A1 (120)	B1 (3.0)	C1 (0.9)
2	A2 (150)	B2 (3.5)	C2 (1.1)
3	A3 (180)	B3 (4.0)	C3 (1.2)

**Table 4:** Orthogonal experimental results.

Experiment number	Factor			Evaluation indicators		
	Anchor cable preload (A)	Anchor cable length (B)	Anchor cable spacing (C)	Roof deformation/mm	Two sides deformation/mm	Plastic zone volume/m^3^
1	1	1	1	50.11	38.13	300.76
2	1	2	2	56.17	47.24	295.86
3	1	3	3	51.18	47.64	295.86
4	2	1	2	55.85	46.66	296.54
5	2	2	3	50.26	46.8	278.46
6	2	3	1	49.25	28.48	264.86
7	3	1	3	50.58	46.24	281.72
8	3	2	1	49.62	36.04	320.35
9	3	3	2	55.54	46.25	289.65

**Table 5:** Mean and range results of each evaluation indicators.

Evaluation indicators	Range	Anchor cable preload (A)	Anchor cable length (B)	Anchor cable spacing (C)
Roof	Mean_1_	52.487	52.180	49.660
	Mean_2_	51.787	52.017	55.853
	Mean_3_	51.913	51.990	50.673
	Range	0.700	0.190	6.193
Two sides	Mean_1_	44.337	43.677	34.217
	Mean_2_	40.647	43.360	46.717
	Mean_3_	42.843	40.790	46.893
	Range	3.690	2.887	12.676
Plastic zone	Mean_1_	297.493	293.007	295.323
	Mean_2_	279.953	298.223	294.017
	Mean_3_	297.240	283.457	285.347
	Range	17.540	14.766	9.976

### Support optimization orthogonal matrix analysis

The evaluation of surrounding rock support effect is a comprehensive evaluation of multi-factor indicators, therefore a three-layer data model is constructed based on the above orthogonal experimental data^32^. The anchor cable prestress, length and spacing are used as the factor layers, and the top plate, the deformation of the two sides and the volume of the plastic zone are used as the test evaluation index layers, as shown in Table 6. The weight matrix of each test evaluation index is obtained respectively, and then the weight matrix that affects the stability of the surrounding rock of the roadway is obtained, and finally the influence weight of different support parameters on the stability of the surrounding rock of the roadway is obtained.The process of solving each matrix is as follows^33^.

1. Experiment evaluation index matrix: If there are *l* factors in the orthogonal experiment, and each factor has *m* levels, the average value of the experimental index at the *j* the level of factor *A*_*l*_ is *k*_*ij*_. If the experimental evaluation index is as large as possible, then *K*_*ij*_ = *k*_*ij*_, and the smaller the experimental evaluation index is, the better, then *K*_*ij*_ = *1/k*_*ij*_ to establish a matrix (10).
  10 $$M = \left[ {\begin{array}{*{20}c} {K_{11} } & 0 & 0 & \cdots & 0 \\ {K_{{{12}}} } & 0 & 0 & \cdots & 0 \\ \cdots & \cdots & \cdots & \cdots & \cdots \\ {K_{1m} } & 0 & 0 & \cdots & 0 \\ 0 & {K_{21} } & 0 & \cdots & 0 \\ 0 & {K_{22} } & 0 & \cdots & 0 \\ \cdots & \cdots & \cdots & \cdots & \cdots \\ 0 & {K_{2m} } & 0 & \cdots & 0 \\ \cdots & \cdots & \cdots & \cdots & \cdots \\ 0 & 0 & 0 & \cdots & {K_{l1} } \\ 0 & 0 & 0 & \cdots & {K_{2} } \\ \cdots & \cdots & \cdots & \cdots & \cdots \\ 0 & 0 & 0 & \cdots & {K_{lm} } \\ \end{array} } \right]$$
2. Factor layer matrix: Let $T_{i} = 1/\sum\limits_{m}^{i = 1} {K_{ij} }$, build the matrix (11).
  11 $$T = \left[ {\begin{array}{*{20}c} {T_{1} } & 0 & 0 & 0 \\ 0 & {T_{2} } & 0 & 0 \\ \cdots & \cdots & \cdots & \cdots \\ 0 & 0 & 0 & {T_{l} } \\ \end{array} } \right]$$
3. Horizontal layer matrix: the range of the factor *A*_*l*_ in the orthogonal experiment is *s*_*i*_, $S_{i} = s_{i} /\sum\limits_{1}^{i = 1} {s_{i} }$ and the matrix (12) is established.
  12 $$S = \left[ {\begin{array}{*{20}c} {S_{1} } \\ {S_{2} } \\ \cdots \\ {S_{l} } \\ \end{array} } \right]$$
4. The weight matrix that affects the value of the experimental evaluation index: w = MTS

13 $$w_{T} { = }\left[ {w_{1} ,w_{2} ,...,w_{m} } \right]$$

**Table 6:** Data structure of orthogonal test.

Level one	Experimental evaluation index			
Second floor	Factor A_1_	Factor A_2_	…	Factor A_*l*_
The third floor	A_11_	A_21_	…	A_*l*1_
	A_12_	A_22_		A*l*_2_
	…	…		…
	A_l*m*_	A_2*m*_		A_*lm*_

Through the calculation of the matrix $w_{l} {\text{ = M}}_{l} T_{l} S_{l}$, the weights of the influence of each factor and each level on the test results can be obtained. According to the weight, the optimal solution and the priority order of the influencing factors can be obtained.

Since the smaller the deformation of the roadway roof, according to the experimental evaluation index weight matrix construction principle *K*_*ij*_ = *1/k*_*ij*_, combined with the data in Table 5, the roof evaluation index matrix *M*_*1*_ is obtained as follows:

14 $$M_{1} = \left[ {\begin{array}{*{20}c} {1/52.487} & 0 & 0 \\ {1/51.787} & 0 & 0 \\ {1/51.913} & 0 & 0 \\ 0 & {1/52.180} & 0 \\ 0 & {1/52.017} & 0 \\ 0 & {1/51.990} & 0 \\ 0 & 0 & {1/49.660} \\ 0 & 0 & {1/55.853} \\ 0 & 0 & {1/50.673} \\ \end{array} } \right]$$

The factor layer matrix *T*_*1*_ can be obtained by formula (11) as follows:

15 $$T_{1} = \left[ {\begin{array}{*{20}c} {{1/156}{\text{.187}}} & 0 & 0 \\ 0 & {{1/156}{\text{.187}}} & 0 \\ 0 & 0 & {{1/156}{\text{.187}}} \\ \end{array} } \right]$$

Combined with formula (12), the horizontal layer matrix *S*_*1*_ can be obtained as shown below.

16 $$S_{1} = \left[ {\begin{array}{*{20}c} {0.7/7.083} \\ {0.19/7.083} \\ {6.193/7.083} \\ \end{array} } \right]$$

Similarly, the experimental evaluation index matrices *M*_*2*_ and *M*_*3*_, the factor layer matrices *T*_*2*_ and *T*_*3*_, and the horizontal layer matrices *S*_*2*_ and *S*_*3*_ are calculated. Combined with formula (13), the weight matrices *w*_*1*_, *w*_*2*_, and *w*_*3*_ that affect the value of the experimental evaluation index can be obtained as follows.

17 $$w_{1} = \left[ \begin{gathered} {0}{\text{.0000121}} \hfill \\ {0}{\text{.0000122}} \hfill \\ {0}{\text{.0000122}} \hfill \\ {0}{\text{.0000033}} \hfill \\ {0}{\text{.0000033}} \hfill \\ {0}{\text{.0000033}} \hfill \\ {0}{\text{.0001127}} \hfill \\ {0}{\text{.0001002}} \hfill \\ {0}{\text{.0001105}} \hfill \\ \end{gathered} \right]\quad w_{2} = \left[ \begin{gathered} {0}{\text{.0000338}} \hfill \\ {0}{\text{.0000369}} \hfill \\ {0}{\text{.0000350}} \hfill \\ {0}{\text{.0000269}} \hfill \\ {0}{\text{.0000271}} \hfill \\ {0}{\text{.0000288}} \hfill \\ {0}{\text{.0001505}} \hfill \\ {0}{\text{.0001103}} \hfill \\ {0}{\text{.0001098}} \hfill \\ \end{gathered} \right]\quad w_{3} = \left[ \begin{gathered} {0}{\text{.000001594}} \hfill \\ {0}{\text{.000001694}} \hfill \\ {0}{\text{.000001596}} \hfill \\ {0}{\text{.000001363}} \hfill \\ {0}{\text{.000001339}} \hfill \\ {0}{\text{.000001409}} \hfill \\ {0}{\text{.000000913}} \hfill \\ {0}{\text{.000000917}} \hfill \\ {0}{\text{.000000945}} \hfill \\ \end{gathered} \right]$$

Since the stability of the roadway cannot be controlled by a single factor, nor can it be investigated with a single index, it is necessary to obtain the total weight matrix of the three inspection indexes. By adding w_1_, w_2_, and w_3_ to obtain the average value of the total weight matrix that affects the stability of the roadway^34,35^. It can be seen from matrix (18) that the optimal solution is A_2_B_3_C_1_, that is, the stability of the surrounding rock of the roadway is the best when the anchor cable prestress is 150KN, the anchor cable length is 4 m, and the anchor cable spacing is 0.9 m.

18 $$w = \frac{{w_{1} + w_{2} + w_{3} }}{3} = \left[ \begin{gathered} {0}{\text{.00001582}} \hfill \\ {0}{\text{.00001693}} \hfill \\ {0}{\text{.00001626}} \hfill \\ {0}{\text{.00001050}} \hfill \\ {0}{\text{.00001056}} \hfill \\ {0}{\text{.00001116}} \hfill \\ {0}{\text{.00008806}} \hfill \\ {0}{\text{.00007047}} \hfill \\ {0}{\text{.00007375}} \hfill \\ \end{gathered} \right] = \left[ {\begin{array}{*{20}c} {A_{1} } \\ {A_{2} } \\ {A_{3} } \\ {B_{1} } \\ {B_{2} } \\ {B_{3} } \\ {C_{1} } \\ {C_{2} } \\ {C_{3} } \\ \end{array} } \right]$$

## Numerical simulation analysis of long and short anchor cable combination support

**Figure 6:**
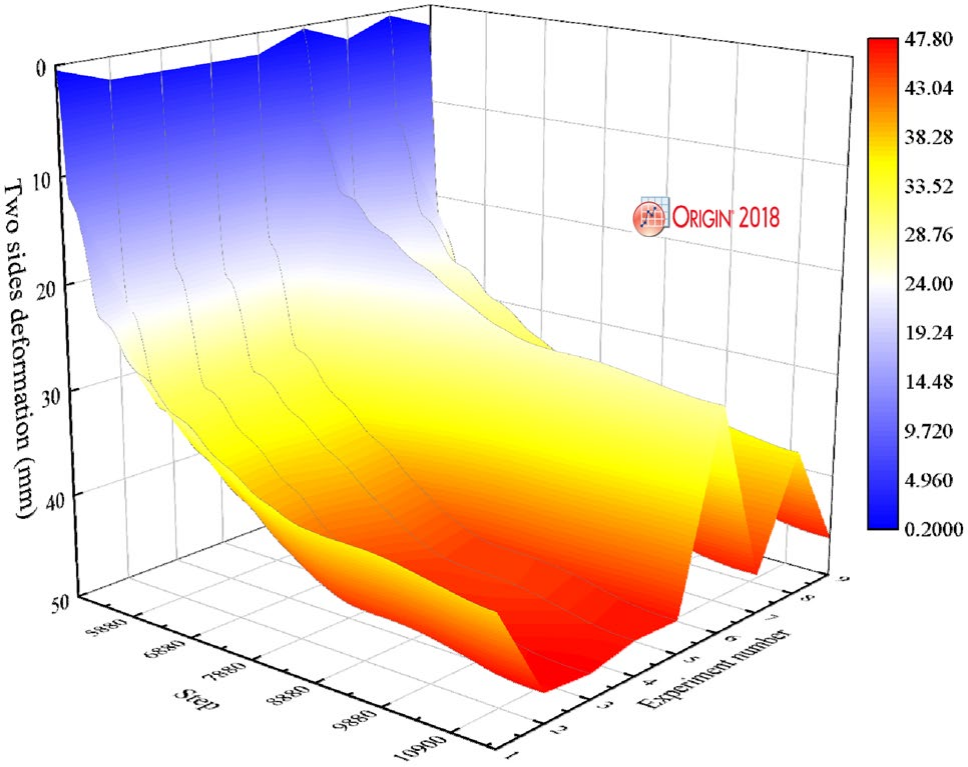
Deformation law of two sides under different support schemes.

**Figure 7:**
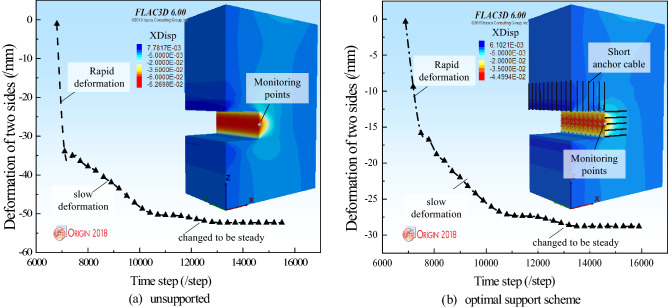
Comparison of displacement fields of two sides of surrounding rock.

**Figure 8:**
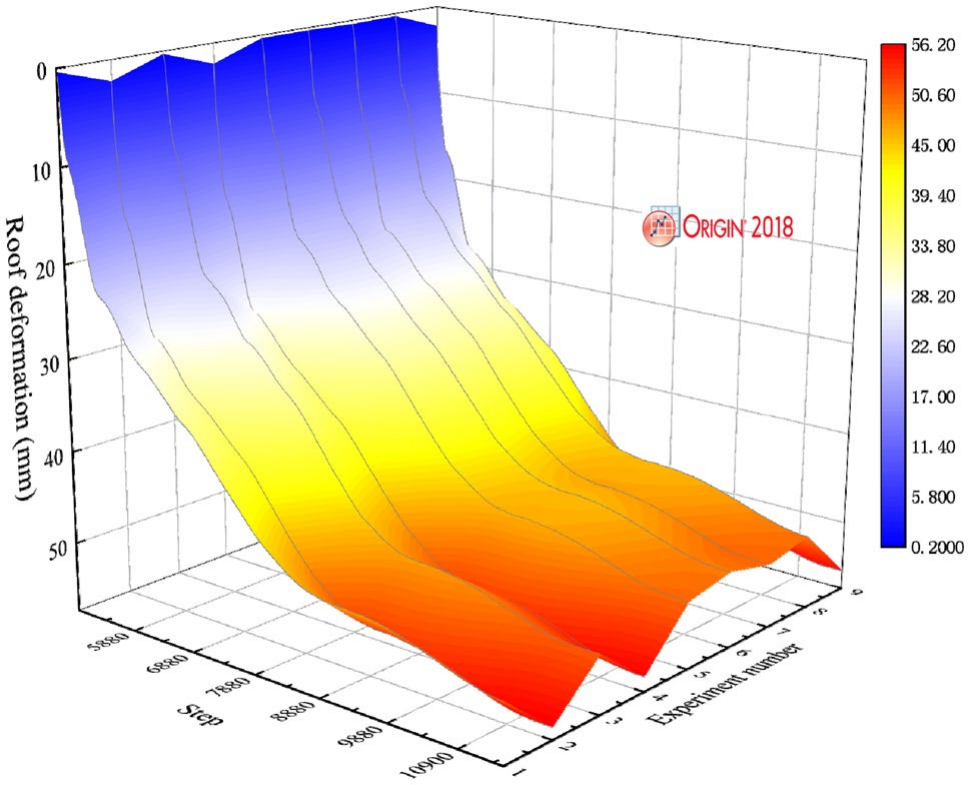
Deformation law of roof under different support schemes.

**Figure 9:**
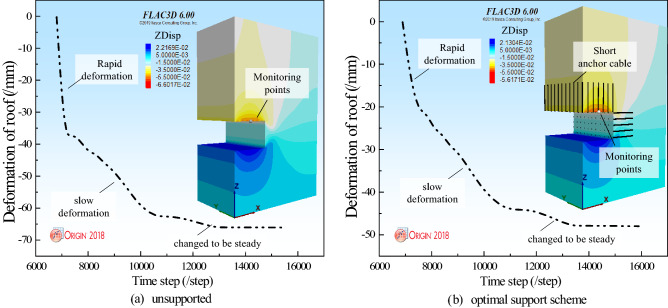
Comparison of displacement fields of two sides of surrounding rock.

**Figure 10:**
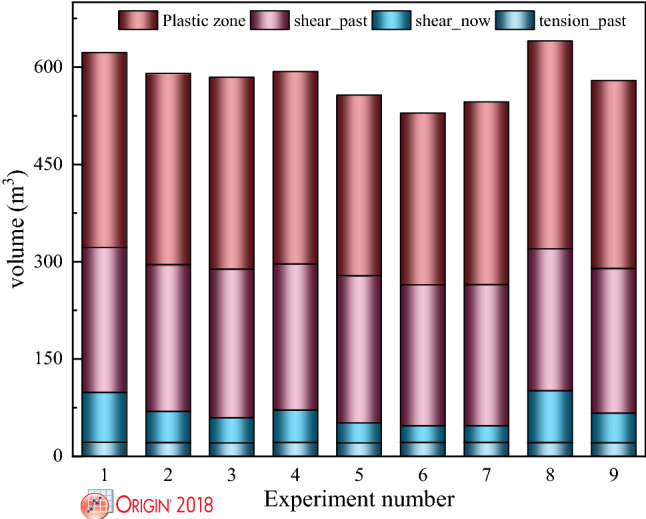
Volume variation law of plastic zone under different support schemes.

**Figure 11:**
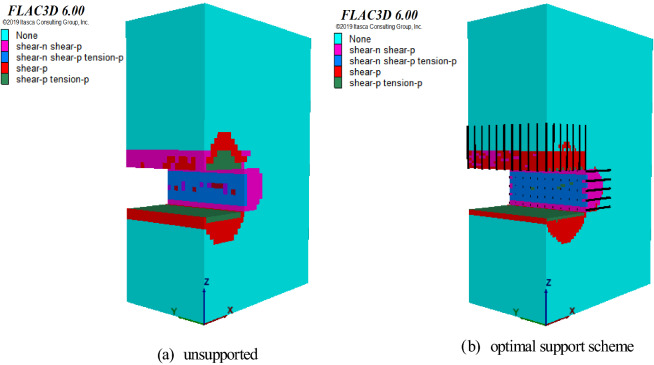
Volume comparison of surrounding rock plastic zone.

Figures 6, 7, 8, 9, 10 and 11 are made by Origin 2018 software^36^ and FLAC3D 6.0 software^37^. Figure 6 illustrates the deformation law of the two sides under different support schemes. In Fig. 6, the deformation law of two sides under the nine support schemes showed rapid deformation-slow deformation-stable deformation. When the support scheme is A_2_B_3_C_1_, the two sides reach the minimum deformation of stability. This is consistent with the results obtained from the orthogonal matrix.

Figure 7 illustrates the comparison of the deformation of the two sides of the surrounding rock under unsupported and optimal support schemes. The monitoring points are arranged on the right side of the surrounding rock. The deformation laws of the two sides of the surrounding rock are obtained. Figure 7a,b show that the deformation on the two sides of the surrounding rock is divided into three developmental stages. When the surrounding rock is just excavated, the surrounding rock enters the stage of rapid deformation. When the surrounding rock excavation releases a part of the energy, the surrounding rock enters the slow deformation stage. The surrounding rock reaches a new equilibrium state, and the deformation of the surrounding rock tends to be stable. Figure 7a shows that when the surrounding rock is not supported, the deformation of the two sides of the surrounding rock reaches 52.6 mm. Figure 7b, when the optimal support scheme supports the surrounding rock, the deformation of the two sides of the surrounding rock reaches 28.8 mm, and the deformation of the two sides is reduced by 45.24%.

Figure 8 illustrates the deformation law of the roof under different support schemes. In Fig. 8, the deformation law of roof under the nine support schemes showed rapid deformation-slow deformation-stable deformation. When the support scheme is A_2_B_3_C_1_, the roof reach the minimum deformation of stability. This is consistent with the results obtained from the orthogonal matrix.

Figure 9 illustrates the comparison of the deformation of the surrounding rock roof under the unsupported and optimal support schemes. The monitoring points are arranged on the roof of the surrounding rock. The deformation law of the roof of the surrounding rock is obtained. Figure 9a,b show that the deformation of the surrounding rock roof is in three stages: rapid deformation, slow deformation, and tending to stability. It can be seen from Fig. 9a that when the surrounding rock is not supported, the two sides of the surrounding rock reach 66 mm. In Fig. 9b, when the optimal support scheme supports the surrounding rock, the roof deformation of the surrounding rock reaches 48 mm, and the roof deformation is reduced by 27.27%.

Figure 10 illustrates the volume variation law of the plastic zone under different support schemes. The failure volume of different states of the surrounding rock was extracted using the self-programmed FISH language. In Fig. 10, when the support scheme is A_2_B_3_C_1_, the total volume of the plastic zone is minimized. Compared with other support schemes, the volume of shear_past, shear_now, and tension_past of the surrounding rock under the group 6 support scheme is the smallest. From the nine support schemes, it can be seen that the surrounding rock is mainly dominated by shear_past.

Figure 11 illustrates the volume comparison of the surrounding rock plastic zone under the unsupported and optimal support schemes. The plastic zone volume display program was written in the FISH language. The volume of shear_now, tension_now, shear_pas, and tension_past was obtained. Figure 11a shows that when the surrounding rock is not supported, the volume values of the four types of damage to the surrounding rock are 227.544, 7.36809, 511.379, and 95.7342, respectively. Figure 11b shows that when the optimal scheme supports the surrounding rock, the volume of the four types of damage to the surrounding rock are 218.453, 2.3168, 442.316, and 43.4006, respectively, decreasing by 3.99%, 67.9%, 13.5%, and 54.66%. After adopting the optimal support scheme, the failure volume values of tension_now and tension_past of the surrounding rock are significantly reduced.

## Conclusion

1. The structural model and roof mechanics model of the top coal roadway in the thick seam are established. Based on the relationship between the radius of curvature and the bending moment of the beam, the maximum bending moment, and ultimate tensile strength of the beam, the deformation coefficient *T*_*K*_ is defined. According to the ratio of deformation rate between *T*_*K*_ and the beam, the deformation mode of the roof of the roadway supported by the top coal is judged, and the conditions for judging the stability of the roadway roof under the two deformation conditions are obtained.
2. Based on the deformation and failure characteristics of the top coal roadway in the thick seam, the coal and rock mass is severely broken, the roof is deformed greatly, and the top coal and the side coal are integrated, high prestressed long and short anchor cable double arch bearing structure control technology is proposed.
3. This paper proposes an orthogonal matrix analysis method for optimization of roadway support parameters in thick coal seam supporting top coal, which can significantly reduce the amount of support plan analysis, realize comprehensive evaluation of multi-factor and multi-support effects of roadway support, and obtain the optimal roadway support plan. Compared with the surrounding rock of the roadway without support, the deformation of the roof is reduced by 27.27%, the deformation of the two sides is reduced by 45.24%, and the tensile failure volume value is reduced by 54.66%. The top coal roadway in the thick coal seam has been effectively controlled, guaranteeing for the high production and high efficiency of the mine (Supplementary information S1).

## Supplementary Information

Supplementary Information.

## Data Availability

The data used to support the findings of this study are included within the article.
